# How People’s COVID-19 Induced-Worries and Multiple Environmental Exposures Are Associated with Their Depression, Anxiety, and Stress during the Pandemic

**DOI:** 10.3390/ijerph20166620

**Published:** 2023-08-21

**Authors:** Jianwei Huang, Mei-Po Kwan, Lap Ah Tse, Sylvia Y. He

**Affiliations:** 1 Institute of Space and Earth Information Science, The Chinese University of Hong Kong, Shatin, Hong Kong, China; jianwei.huang@link.cuhk.edu.hk (J.H.); shelly@cuhk.edu.hk (L.A.T.); 2 Department of Geography and Resource Management, The Chinese University of Hong Kong, Shatin, Hong Kong, China; sylviahe@cuhk.edu.hk; 3 Division of Occupational and Environmental Health, JC School of Public Health and Primary Care, The Chinese University of Hong Kong, Shatin, Hong Kong, China

**Keywords:** COVID-19 pandemic, COVID-19-induced worries, mental health, multiple environmental exposures, mobility

## Abstract

This study investigates how people’s perceived COVID-19 risk, worries about financial hardship, job loss, and family conflicts, and exposures to greenspace, PM_2.5_, and noise (in people’s residential neighborhoods and daily activity locations) are related to their depression, anxiety, and stress during the COVID-19 pandemic. Using a two-day activity-travel diary, a questionnaire, and real-time air pollutant and noise sensors, a survey was conducted to collect data from 221 participants living in two residential neighborhoods of Hong Kong during the COVID-19 pandemic. Linear regression was conducted to explore the relationships. Significant associations between people’s COVID-19-related worries and exposures to grassland and PM_2.5_ with depression, anxiety, and stress were found in the results. These associations with depression, anxiety, and stress vary depending on people’s demographic attributes. These results can help direct the public authorities’ efforts in dealing with the public mental health crisis during the COVID-19 pandemic.

## 1. Introduction

The COVID-19 pandemic, caused by the novel severe acute respiratory syndrome coronavirus 2 (SARS-CoV-2), has caused a huge health burden around the world since 2020. To reduce the transmission of COVID-19, governments around the world designed and implemented drastic non-pharmaceutical interventions (e.g., social distancing). A common assumption underlying these measures is that people’s risky social interactions (e.g., face-to-face contact) would be reduced due to a decrease in their daily mobility [1,2,3,4]. However, the non-pharmaceutical interventions have also resulted in major disruptions to public health infrastructure and societal norms. Moreover, these measures may have detrimental effects on people’s mental well-being because of their impacts on people’s economic status and fear of being infected [5,6].

Previous studies have found that the COVID-19 pandemic and the mitigation measures induced enormous social and economic impacts (e.g., financial hardship, job loss, and family conflicts), which worsened people’s existing depression, anxiety, and stress. For instance, using an online survey dataset collected in May 2020, Gadermann et al. [7] revealed that parents with children younger than 18 years old living at home reported worse mental health due to potential family conflicts during the pandemic than adults without children younger than 18 years old living at home. Posel et al. [8] indicated that adults who retained paid jobs reported significantly lower depression and anxiety scores than adults who lost their jobs during the COVID-19 lockdown in South Africa, based on a National Income Dynamics-Coronavirus Rapid Mobile Survey (NIDS-CRAM) in 2020. Using the United Kingdom Household Longitudinal Study (UKHLS) dataset, Pierce et al. [9] reported that significant clinical mental distress rose from 18.9% in 2018–19 to 27.3% in April 2020 among the general population. In Brazil, Goularte et al. [10] demonstrated that the most common psychiatric symptoms of people’s mental health problems include anxiety, depression, anger, somatic symptoms, and sleep problems among the general population based on an online web-based survey dataset during the COVID-19 pandemic. Thus, evidence from different countries indicated that the enormous social and economic impacts of the COVID-19 pandemic (e.g., financial hardship, job loss, and family conflicts) have significantly increased people’s mental health issues. Moreover, studies have observed that the mental health consequences caused by the COVID-19 pandemic are not equal: the impacts of the COVID-19 pandemic on people’s mental health would be different over space and across various sociodemographic groups [11,12].

Although previous studies have enhanced our understanding of how COVID-19-induced worries (e.g., perceived COVID-19 risk, worries about financial hardship, job loss, and family conflicts) may have a harmful effect on their mental health, they did not examine the combined associations between COVID-19-induced worries and multiple environmental exposures with mental health. Specifically, people’s mental health would also be affected by their exposure to greenspace, air pollution, and noise in their daily lives. Greenspace exposure can mitigate the negative impacts of people’s exposure to environmental stressors while restoring their attention and facilitating physical activities or social cohesion [13,14,15]. For instance, using Dutch national survey data, Klompmaker et al. [16] found that people’s exposure to greenspace in their residential neighborhoods can significantly improve mental health, while the beneficial impacts of greenspace may be underestimated if other environmental factors (e.g., air pollution or noise) are ignored. Meanwhile, Ribeiro et al. [17] revealed that people’s exposures to greenspace had a positive association with better mental health outcomes during the COVID-19 pandemic based on online survey data in Portugal and Spain. The great potential benefits of greenspace exposure on people’s mental health have also been observed in the United Kingdom [18], the United States [19,20], Belgium [21], Greece [22], and France [23].

In contrast to greenspace, air pollution and noise are regarded as pervasive environmental stressors relevant to people’s mental health. Specifically, air pollution was found to affect people’s mental health through its impact on neuroinflammation and pulmonary oxidative stress [24,25,26]. Meanwhile, the impact of noise on people’s mental health manifests indirectly through people’s responses to the sound environment (e.g., sleep disturbance) [27,28]. Therefore, people’s exposure to air pollution and noise in their daily lives could worsen mental health conditions such as depression, anxiety, and stress [29]. For instance, using residential noise and air pollution data estimated through land use regression models, Dzhambov et al. [25] found that individuals’ air pollution and noise exposures are significantly associated with their mental health and well-being (e.g., higher levels of air pollution and noise exposures are significantly associated with worse mental health). Tao et al. [30] also observed that air pollution and noise exposures could induce people’s momentary stress in certain microenvironments (e.g., rush hours and traveling by public transit).

Although previous studies have examined how multiple environmental exposures would affect people’s mental health, they may generate misleading results due to the uncertain geographic context problem (UGCoP) and the neighborhood effect averaging problem (NEAP), which suggest that the assessments of people’s mobility-dependent exposure to greenspace, air pollution, and noise may be biased if people’s daily mobility is ignored [31,32,33,34]. Specifically, previous studies tend to use a traditional residence-based approach to estimate people’s environmental exposures [35,36,37]. A major assumption underlying the traditional residence-based approach is that people’s health outcomes are mainly affected by their residential neighborhood environments. However, most people typically have to conduct daily activities (e.g., education and work) in areas outside their residential neighborhoods. They are thus exposed to multiple environments outside of their residential neighborhoods [38,39,40]. Therefore, ignoring people’s daily mobility can result in biased estimates of people’s environmental exposures, which in turn may generate misleading conclusions.

**Figure 1:**
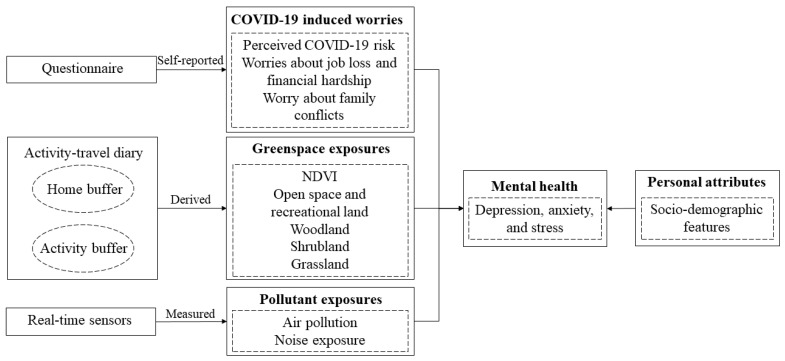
Conceptual framework.

This study thus seeks to investigate the combined associations between COVID-19-induced worries (i.e., perceived COVID-19 risk, worries about financial hardship, job loss, and family conflicts) and exposures to multiple environmental factors (i.e., greenspace, air pollution, and noise) with depression, anxiety, and stress during the COVID-19 pandemic, both in the residential neighborhood and in people’s daily activity locations. Figure 1 presents the conceptual framework of the study. Briefly, we conducted a survey with participants who lived in two typical residential neighborhoods of Hong Kong (i.e., Sham Shui Po with a high COVID-19 risk and Tin Shui Wai with a low COVID-19 risk) during the pandemic. The survey employed real-time air pollution and noise sensors, a two-day activity and travel diary, and a questionnaire to collect data from participants. The data include participants’ COVID-19-induced worries (i.e., perceived COVID-19 risk, worries about financial hardship, job loss, and family conflicts), residential and daily activity locations, and real-time PM_2.5_ and noise exposures. In addition, we also collected a Normalized Difference Vegetation Index (NDVI) dataset and a land-use dataset to assess participants’ exposures to different types of greenspace in their residential neighborhoods and daily activity locations. Using the dataset and measurements, we applied multiple linear regression to separately explore the combined associations between COVID-19-induced worries and multiple environmental exposures with depression, anxiety, and stress. Lastly, we explored whether these associations differ by participants’ demographic attributes.

## 2. Dataset and Methods

### 2.1. Study Design and Sampling

A survey was conducted in Sham Shui Po (SSP) and Tin Shui Wai (TSW) in Hong Kong from April 2021 to September 2021, which represent two typical neighborhoods in the city during the pandemic. Note that SSP is a high-risk neighborhood that suffered repeated COVID-19 outbreaks from January 2020 to May 2021 due to its socio-demographic and built-environment characteristics [41,42]. Conversely, TSW is a low-risk neighborhood for COVID-19 from January 2020 to May 2021 [42,43]. The government persistently implemented a “zero-COVID” strategy from January 2020 to March 2022. The control measures include border control, social distancing, restricted dine-in, and restrictions or closures of clubs, bars, and public facilities (e.g., schools and museums). Therefore, although the survey was conducted after the peak of COVID-19 outbreaks in the city, people’s daily lives were still affected due to the control measures.

We recruited 221 residents (aged 18–64 years) from the two neighborhoods based on a stratified sampling approach. The samples of the two neighborhoods represent their respective populations well, and a more elaborate description of the participants’ sociodemographic profile versus the census demographics of the two neighborhoods is provided in Kan et al. [43] and Huang and Kwan [44]. It should be noted that people’s mobility in Hong Kong hardly declined during the time of the survey, despite the control measures (e.g., social distancing) still being effective. For instance, people’s daily mobility to grocery outlets (e.g., supermarkets, cook food markets, farmers markets, and pharmacies) increased by over 10% when compared with the baseline (i.e., the mean value of mobility in January 2020) [45].

During the survey, each participant was required to carry a portable air pollutant sensor (logged at one-second intervals), a portable noise sensor (logged at 30-s intervals), and a two-day activity-travel diary over two continuous days (i.e., a weekday and a weekend day). We used the sensors and the activity-travel diary to collect the participants’ real-time exposure to PM_2.5_ concentrations and noise, the places they visited, and the activity durations on the two survey days. Additionally, the participants were also required to complete a questionnaire in a face-to-face briefing session, which solicits their personal and household socioeconomic attributes (e.g., age, biological sex, household income, and so on), COVID-19-induced worries during the pandemic, and subjective evaluations of one’s mental health status (e.g., depression, anxiety, and stress). The survey protocol and questionnaire were reviewed and approved by the Survey and Behavioural Research Ethics Committee (SBRE) of the Chinese University of Hong Kong. Informed consent was obtained from all participants before data were collected from them.

### 2.2. Depression, Anxiety, and Stress as Outcomes

In this study, the outcomes include two different variables: (1) participants’ depression and anxiety, and (2) stress. Participants’ depression and anxiety were measured by the well-established Patient Health Questionnaire-4 (PHQ-4) [46]. Table S1 (Supplementary Materials) presents the PHQ-4 items, which include 4 different questions. The participants were asked to answer each question on a 6-point scale. The total score for depression and anxiety was obtained by adding together the scores of each of the 4 response items, and it ranges from 4 to 24. A higher total score indicates more severe depression and anxiety. The Cronbach’s alpha index of the depression and anxiety items is 0.89, indicating excellent internal consistency. 

Participants’ stress levels were measured by their self-reported frequency of several symptoms over the past year. Table S2 presents the 4 items of symptoms, which include options on a 6-point scale. We expected that people’s stress levels could be represented by using a one-factor structure underlying the 4 symptom items. Thus, exploratory factor analysis (EFA) using principal axis factoring and oblique promax rotation was applied to evaluate and extract the one-factor structure. We applied Kaiser’s rule to select the factor solution (i.e., the factor solution should have eigenvalues > 1). The results indicated a clear one-factor solution (eigenvalue = 1.58) that includes 3 items (i.e., excluding an item with a factor loading < 0.30). Table S2 also shows the factor loadings. A higher total score indicates more severe stress.

### 2.3. COVID-19-Induced Worries

People’s COVID-19-induced worries include their residence-based and mobility-based perceived COVID-19 risk and worries about job loss, financial hardship, and family conflicts during the pandemic. Table S3 presents questions about COVID-19-induced worries. Each question was quantified based on a 6-point scale, and a higher score indicates more severe worry. People’s worries about financial hardship and job loss were summed to produce a total score. The Cronbach’s alpha index of worries about financial hardship and job loss items was 0.97, indicating excellent internal consistency.

### 2.4. Greenspace Exposure Assessment

To assess participants’ greenspace exposure, we collected a Normalized Difference Vegetation Index (NDVI) dataset and a land-use dataset. The NDVI dataset has a spatial resolution of 6 m × 6 m and was derived from SPOT-7 Satellite images in 2017. The land-use dataset was compiled using multiple data sources (e.g., satellite images dated December 2019, in-house survey information of the Planning Department up to end-2019) and provided by the Hong Kong Planning Department in 2020. The land-use dataset has a spatial resolution of 10 m × 10 m and includes 4 different types of greenspace: (1) open space and recreational land, which include parks, stadiums, playgrounds, and recreational facilities; (2) woodland; (3) shrubland; and (4) grassland [44]. It should be noted that the original NDVI values range from −1 to 1, and we excluded negative values since they represent non-greenspace (e.g., water bodies). In addition, the NDVI also included private greenery (e.g., private gardens) and street greenery in different types of land-use areas (e.g., buildings and along roads), while the land-use dataset does not. Dissimilar to the NDVI, different types of greenspace in the land-use dataset are more associated with publicly accessible greenspace (e.g., community parks and country parks) for urban residents. The advantages of the land-use dataset also include the possibility of distinguishing between different types of greenspace.

Greenspace exposure was assessed using buffers with a 500 m radius (i.e., walking distance < 10 min) for both residential and activity locations reported by the participants. Specifically, we use two different measurements to assess participants’ greenspace exposure: (1) residence-based greenspace exposure approach, which assessed participants’ different types of greenspace (i.e., NDVI, open space and recreational land, woodland, shrubland, and grassland) within 500 m buffer area around their home locations; (2) mobility-based greenspace exposure approach, which assessed participants’ different types of greenspace within 500 m buffer area around their daily activity locations. A more comprehensive description of the residence-based and mobility-based greenspace exposure measurements is provided in Huang and Kwan [40].

We further applied EFA using principal axis factoring and oblique promax rotation to explore a potential factor structure underlying the different types of estimated greenspace exposures. The Kaiser’s rule was used to select the factor solution (i.e., the factor solution should have eigenvalues > 1). Specifically, we expected there to be a potential factor underlying the different types of estimated greenspace exposures that would present people’s combined greenspace exposure. Note that people’s exposure to open space and recreational land was excluded since it has nonsignificant correlations with other types of greenspace exposures (see Figure S1). The EFA results clearly indicated a one-factor solution under 4 items (i.e., woodland, shrubland, grassland, and NDVI) for residence-based greenspace exposure (eigenvalue = 2.23) and mobility-based greenspace exposure (eigenvalue = 1.72). Table S4 shows brief statistical descriptions and factor loadings of the items. 

### 2.5. PM_2.5_ and Noise Exposures

As Section 2.1 mentioned, real-time spatiotemporal data on PM_2.5_ concentrations and noise levels were measured using portable sensors, which were carried by the participants over two continuous days. The real-time measurements can simultaneously monitor PM_2.5_ concentrations and noise levels at a high spatiotemporal resolution (i.e., every second for PM_2.5_ and every 30 s for noise level). To improve the accuracy of the PM_2.5_ concentrations recorded by the air pollution sensors, we developed a machine-learning-based calibration model using the colocation analysis method. The calibration model adjusted the PM_2.5_ concentrations recorded by the portable air pollutant sensors to align them with the data recorded by a professional-grade DustTrak DRX Aerosol Monitor 8533 sensor and obtain a strong correlation (R^2^ range from 0.90 to 0.95). More information about the machine-learning-based calibrated model can be found elsewhere [47]. The mean values of the calibrated PM_2.5_ concentrations over the two continuous days were used to represent participants’ PM_2.5_ exposure levels. 

Regarding the real-time noise data, a professional-grade CEM SC-05 Sound Level Calibrator was employed to calibrate each portable noise sensor before they were distributed to the participants. The calibrated portable noise sensors have a measurement range of 30–130 dBA with an accuracy of <1.5 dBA error, which meets IEC61672 Type 2 Sound Level Meter standards. With the recorded noise data, we further used equivalent A-weighted sound pressure levels to evaluate the sound levels in the daytime (10:00 am–18:00 pm) and nighttime (00:00 am–7:00 am). A-weighted equivalent sound pressure level refers to the average value of A sound level according to the sound energy for a certain period, which has been widely used in previous studies to measure individuals’ noise exposures [30].

### 2.6. Statistical Analyses

We first used descriptive statistics to explore the range and distribution of participants’ depression, anxiety, stress, and worries about financial hardship, job loss, and family conflicts during the pandemic. Paired sample *t*-test was then used to assess the statistical significance of the difference between people’s residence-based and mobility-based perceived COVID-19 risk and greenspace exposures and to test the difference in noise exposure between daytime and nighttime. Then, we used Spearman correlations to explore the bivariate associations among the main variables. 

Further, we used separate linear regression models to explore the combined associations between people’s perceived COVID-19 risk, worries about financial hardship, job loss, family conflicts, and exposures to greenspace, air pollution, and noise with depression, anxiety, and stress during the pandemic, both in the residential neighborhood and in people’s daily activity locations. Specifically, we first focused on people’s depression and anxiety in Models 1–6, while residence-based environmental exposures were included in Models 1–3 and mobility-based environmental exposures were included in Models 4–6. Then, we focused on people’s stress in Models 7–12, while residence-based environmental exposures were included in Models 7–9 and mobility-based environmental exposures were included in Models 10–12. In Models 1, 4, 7, and 10, we examined the associations between people’s depression, anxiety, and stress with their residence- and mobility-based perceived COVID-19 risk and worries about financial hardship, job loss, and family conflicts. We further included people’s residence- and mobility-based exposures to greenspace, air pollution, PM_2.5_, daytime noise, and nighttime noise exposures in Models 2, 5, 8, and 11. In the full models (i.e., Models 3, 6, 9, and 12), we constructed the interaction terms by combining people’s exposures to greenspace, open space, and recreational land with their perceived COVID-19 risk and worries about financial hardship, job loss, and family conflicts to observe if the effects of COVID-19-induced worries on people’s depression, anxiety, and stress were influenced by greenspace. It should be noted that all models control for participants’ sociodemographic attributes, including sex, age, education attainment, employment status, marital status, monthly household income, workplaces (e.g., Kowloon or Hong Kong Island), residential neighborhoods (e.g., TSW or SSP), housing types (e.g., private housing or social housing), homeownership (e.g., rented or owned their residential house), and monthly household rent/mortgage payment. Before fitting the models, the variance inflation factors (VIFs) were used to assess multicollinearity among the main variables. All VIFs of independent variables are less than 8.0, indicating a low probability of multicollinearity. We use the Akaike information criterion (AIC) and adjusted R^2^ to assess the performance of these models. 

In addition, we also explored whether the combined associations differ by people’s demographic attributes. Specifically, we performed stratified analyses based on participants’ demographic attributes (i.e., sex, marital status, monthly household income, residential neighborhoods, and housing types). The analysis was conducted in R version 4.1.0.

## 3. Results

### 3.1. Descriptive Statistics

A total of 217 participants (107 from Sham Shui Po [SSP] and 110 from Tin Shui Wai [TSW]) were finally included in our study. We excluded participants with invalid data (i.e., missing data in the two-day activity diary, PM_2.5_, or noise records). Table 1 shows participants’ sociodemographic attributes in the two neighborhoods. It indicates that participants from the two neighborhoods represent their respective populations well [44,45]. The mean scores of depression and anxiety for participants in the SSP and TSW are 13.80 (standard deviation [SD]: 3.82) and 13.75 (SD: 4.11). The mean score of stress for participants is 15.21 (SD: 2.92) in SSP and 14.94 (SD: 3.20) in TSW. Furthermore, the mean scores of worry about family conflicts for participants in SSP and TSW are 3.23 (SD: 1.30) and 2.85 (SD: 1.32), while the mean scores of worries about financial hardship and job loss for participants are 7.07 (SD: 2.81) in SSP and 5.55 (SD: 1.32) in TSW. The mean value of residence-based perceived COVID-19 risk for participants is 3.37 (SD: 0.95) in SSP and 2.94 (SD: 0.77) in TSW, and the mean value of mobility-based perceived COVID-19 risk for participants is 2.48 (SD: 0.88) in SSP and 2.50 (SD: 0.90) in TSW.

Table 2 presents the summary statistics of participants’ multiple environmental exposures. Specifically, the differences between residence-based and mobility-based exposures to greenspace, open space, and recreational land are statistically significant (*p* < 0.05). The mean value of PM_2.5_ is 12.43 (SD:5.57) (ug/m^3^). In addition, participants’ exposures to daytime and nighttime noise are significantly different (*p* < 0.05).

### 3.2. Bivariate Analysis

**Figure 2:**
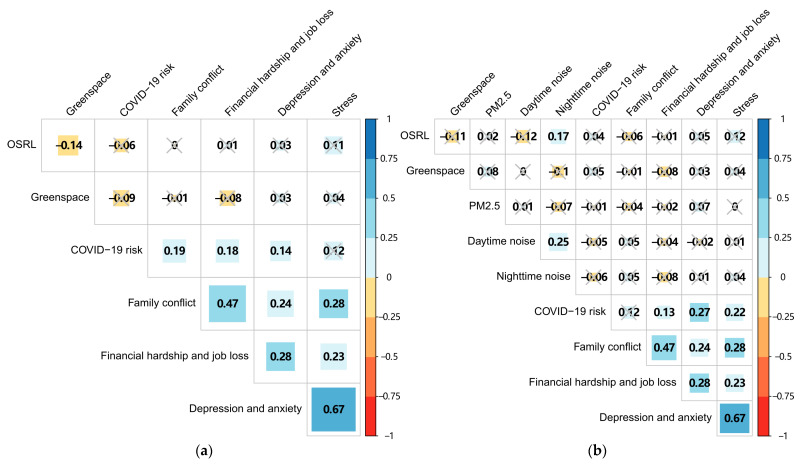
Correlation matrix of (**a**) residence-based and (**b**) mobility-based exposures based on Spearman correlation coefficients. Note that Cells marked with “X” refer to insignificant correlations (i.e., *p* > 0.05). OSRL refers to open space and recreational land.

Figure 2 shows the Spearman correlations among participants’ COVID-19-induced worries, multiple environmental exposures, and their scores of depression, anxiety, and stress. First, participants’ depression and anxiety have a significant positive correlation with stress (r = 0.64, *p* < 0.05). Second, participants’ depression and anxiety are significantly and positively correlated with their residence- and mobility-based perceived COVID-19 risk (r = 0.14 and 0.27, *p* < 0.05), worry about family conflicts (r = 0.24, *p* < 0.05), and worries about financial hardship and job loss (r = 0.28, *p* < 0.05). Meanwhile, participants’ stress is also significantly and positively correlated with their residence- and mobility-based perceived COVID-19 risk (r = 0.14 and 0.20, *p* < 0.05), worry about family conflicts (r = 0.32, *p* < 0.05), and worries about financial hardship and job loss (r = 0.32, *p* < 0.05). Correlations between participants’ depression, anxiety, and stress with all types of greenspace, noise, and PM_2.5_ exposures are largely insignificant.

In addition, participants’ residence-based exposure to open space and recreational land is significantly and negatively correlated with their residence-based exposure to greenspace (r = −0.14, *p* < 0.05). Regarding participants’ mobility-based exposures, open space and recreational land are positively correlated with nighttime noise (r = 0.17, *p* < 0.05). Daytime noise is positively correlated with nighttime noise (r = 0.25, *p* < 0.05). The correlations between greenspace, noise, and PM_2.5_ exposures are insignificant. Correlations between COVID-19-induced worries with greenspace, open space, recreational land, noise, and PM_2.5_ exposures are largely insignificant.

### 3.3. Regression Analysis

Models 1–6 focus on people’s depression and anxiety. Table 3 reports the regression models of the associations between participants’ residence- and mobility-based multiple environmental exposures and COVID-19-induced worries and their depression and anxiety. We find that people’s perceived COVID-19 risk is significantly associated with their depression and anxiety for both residence- and mobility-based models (i.e., Models 1–6). In addition, people’s mobility-based perceived COVID-19 risk plays a more important role than their residence-based perceived COVID-19 risk in depression and anxiety: the estimated coefficient of mobility-based perceived COVID-19 risk is 0.22–0.23 in Models 4–6, while the estimated coefficient of residence-based perceived COVID-19 risk is 0.12–0.15 in Models 1–3. Our results also indicate that people’s worries about financial hardship and job loss (Coef. = 0.19–0.23, *p*-value < 0.01, Models 1–6) are significantly associated with people’s depression and anxiety for both residence- and mobility-based models (i.e., Models 1–6). The effects of people’s perceived COVID-19 risk and worries about financial hardship and job loss on their depression and anxiety are not influenced by open space, recreational land, or greenspace. Meanwhile, open space, recreational land, and greenspace are not significantly associated with people’s depression and anxiety in both residence- and mobility-based models. In Model 5, PM_2.5_ is a significant environmental factor associated with higher levels of people’s depression and anxiety (Coef. = 0.11, *p*-value < 0.05).

Models 7–12 focus on people’s stress. Table 4 reports the regression models of the associations between participants’ residence- and mobility-based multiple environmental exposures and COVID-19-induced worries and stress. The results indicate that people’s worries about family conflicts (Coef. = 0.16–0.21, *p*-value < 0.01) and worries about financial hardship and job loss (Coef. = 0.13–0.18, *p*-value < 0.05) are significant for both residence- and mobility-based models (i.e., Models 7–12). In the mobility-based models (i.e., Models 10–12), participants’ perceived COVID-19 risk (Coef. = 0.17–0.18, *p*-value < 0.01, Models 10–12) has a significant and positive association with their stress. The effects of people’s perceived COVID-19 risk and COVID-19-induced worries on their stress are not influenced by open space, recreational land, or greenspace. In the residence- and mobility-based models, open space and recreational land (Coef. = 0.14–0.15, *p*-value < 0.05, Models 8, 11, and 12) are also significantly associated with people’s stress. Lastly, PM_2.5_, daytime noise, and nighttime noise are not significantly associated with people’s stress. 

### 3.4. Stratified Analysis

**Figure 3:**
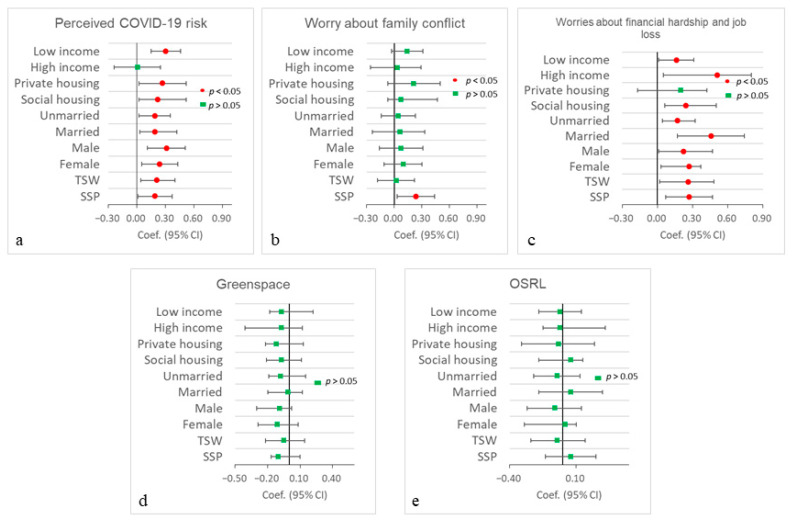
Associations of COVID-19-induced worries, grassland, and PM_2.5_ exposures with people’s depression and anxiety in mobility-based linear regression models across different groups. Private housing includes private houses and tong lau/subdivided units. Unmarried includes single, widowed, or divorced. (**a**) Preceived COVID-19 risk; (**b**) Worry about family conflict; (**c**) Worries about financial hardship and job loss; (**d**) Greenspace; (**e**) OSRL.

**Figure 4:**
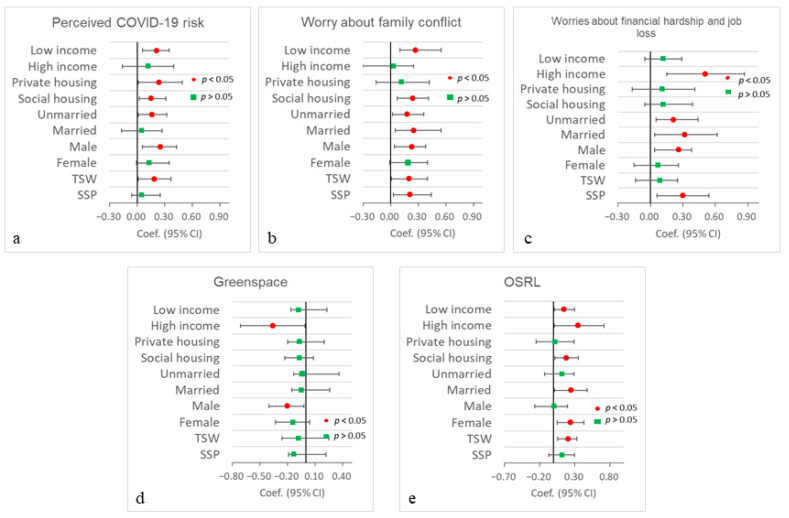
Associations of COVID-19-induced worries, grassland and PM_2.5_ exposures with people’s stress in mobility-based linear regression models across different groups. Private housing includes private houses and tong lau/subdivided units. Unmarried includes single, widowed, or divorced. (**a**) Preceived COVID-19 risk; (**b**) Worry about family conflict; (**c**) Worries about financial hardship and job loss; (**d**) Greenspace; (**e**) OSRL.

Tables S5 and S6 (Supplementary Materials) present the performance of the regression models for examining the combined associations between participants’ multiple environmental exposures and COVID-19-induced worries with their depression, anxiety, and stress across different groups. Figure 3 and Figure 4 show the regression coefficients according to the full mobility-based models. Participants’ mobility-based perceived COVID worries about financial hardship and job loss play the most important role in their depression, anxiety, and stress. Specifically, for most social groups (except for the private housing group), worries about financial hardship and job loss have significant and positive associations with people’s depression, anxiety, and stress. The high-income group has the highest regression coefficients when compared with those of other groups for depression, anxiety, and stress. Participants’ mobility-based perceived COVID-19 risk has significant positive associations with their depression and anxiety for most of the groups (except for the high-income group), while mobility-based perceived COVID-19 risk has significant positive associations with stress for the low-income, private housing, social housing, unmarried, male, and TSW groups. Worrying about family conflicts has an insignificant association with depression and anxiety across different groups (except for the SSP group), while it is significantly and positively associated with stress for most of the groups (except for the high-income, private housing, and female groups). 

In addition, greenspace, open space, and recreational land have insignificant associations with depression and anxiety across all groups. Greenspace has a significant negative association with stress for high-income and male groups, while open space and recreational land have a significant positive association with stress for high-income, low-income, social housing, married, female, and TSW groups. Lastly, Tables S7–S11 report all the regression models of associations between participants’ residence- and mobility-based multiple environmental exposures and COVID-19-induced worries and their depression, anxiety, and stress across different social groups.

## 4. Discussion

### 4.1. Main Findings

This study seeks to investigate the combined associations between people’s COVID-19-induced worries and multiple environmental exposures with depression, anxiety, and stress, both in people’s residential neighborhoods and at their daily activity locations. Our regression models revealed significant positive associations between people’s worries about financial hardship and job loss and depression, anxiety, and stress in all residence-based and mobility-based models. Meanwhile, people’s perceived COVID-19 risk has a significant positive association with depression and anxiety in all residence-based and mobility-based models, while it also has a significant positive association with stress in all mobility-based models. People’s worry about family conflicts has a significant association with stress in all residence-based and mobility-based models. No significant association was observed between people’s worry about family conflicts and depression and anxiety in all residence-based and mobility-based models. 

In addition, in our regression analyses, the associations between greenspace, open space, recreational land, and PM_2.5_ with depression and anxiety were not significant in all residence-based models or the minimal mobility-based models. The association between PM_2.5_ and depression and anxiety was significant in the full mobility-based model. Meanwhile, no significant associations were observed between daytime and nighttime noise and depression, anxiety, or stress. In addition, open space and recreational land have a significant positive association with stress in residence- and mobility-based models. Our results also indicate that these estimated coefficients between people’s COVID-19-induced worries and multiple environmental exposures with depression, anxiety, and stress are different between the residence-based and mobility-based models. Finally, we observed that the combined associations differ by people’s demographic attributes.

### 4.2. Comparisons with Previous Studies and Implications

Our findings suggest that high perceived COVID-19 risk and worries about financial hardship, job loss, and family conflicts are related to people’s poor mental well-being, which corroborates prior work linking COVID-19-induced worries with poor mental well-being [7,48,49,50]. People’s worries about financial hardship and job loss are associated with more depression, anxiety, and stress symptoms. This association may indicate that people’s worries about financial hardship and job loss are the most important risk factors for depression, anxiety, and stress during the COVID-19 pandemic. In addition, the demographic differences indicate that for almost all social groups (except people who have private housing), worries about financial hardship and job loss are significantly associated with depression, anxiety, and stress, and high-income and married groups have higher coefficients than the counterpart groups. Previous studies have indicated that mental health problems are a major public health crisis in Hong Kong, and more people in early 2020 will have poor mental health than in 2016–17, while the older and underprivileged groups suffer most [51]. Our results further imply that the mental health crisis may further spread to different social groups (e.g., the high-income group) in Hong Kong due to the persistence of the economic impacts of the COVID-19 pandemic. Hence, policymakers should also seek to address the public mental health crisis resulting from the economic impacts of the pandemic when developing COVID-19 intervention measures. 

As expected, both residence-based and mobility-based perceived COVID-19 risks are significantly associated with depression and anxiety. Meanwhile, mobility-based perceived COVID-19 risk is significantly associated with stress. The fear of being infected in residential neighborhoods and at daily activity locations may curtail people’s routine daily lives and social interactions, which may further lead to poor mental health. Moreover, COVID-19-infected persons may suffer cyberbullying or cyberviolence after their personal information (e.g., age, sex, and residential location) and visited locations are disclosed to the public by the government [4]. Therefore, the potential discrimination and stigma related to COVID-19 might also make people fearful of infection, which can also worsen their mental health. It is worth noting that previous studies have also observed the negative effect of potential discrimination and stigma related to COVID-19 on people’s mental health in Canada [52], Nepal [53], China [54], Spain [55], and the United States [56]. Therefore, policymakers should consider these negative effects on people’s mental health when they are designing mitigation measures (e.g., disclosing COVID-19 patients’ residential and visited locations to the public). On the other hand, scholars and public authorities should pay more attention to people’s geoprivacy worries and their impacts on their mental health.

Further, we found that people’s worries about family conflicts are associated with stress. One of the potential explanations is that people spent more time with their family members during the pandemic due to the closure of schools and stay-at-home orders in Hong Kong. Further, this association is significant for the male group but insignificant for the female group. Similar associations have also been reported by other studies [7]. This sex difference in the association between worry about family conflicts and stress is contrary to pre-pandemic studies, which suggested that females are disproportionately affected by family conflicts [57,58,59]. This result suggests that future research should further explore the sex differences in this association before and after the COVID-19 pandemic. 

Regarding the benefits of greenspace on depression, anxiety, and stress, we only observed a significant association between greenspace and stress for high-income and male groups (see Figure 4). These results are inconsistent with previous studies that revealed the benefits of residence-based greenspace on people’s depression and anxiety [16]. Meanwhile, Roberts and Helbich [60] also reported a significant association between greenspace exposure and depression and anxiety when applying a 50-m buffer around people’s daily GPS trajectories. The differences in these results might be due to the uncertain geographic context problem (UGCoP), which suggests that using different geographically delineated contextual areas to assess people’s environmental exposures may lead to different research findings on the health effects of environmental factors [31,32,34,40]. In addition, our results also suggest that open space and recreational land have a significant positive association with stress in the residence- and mobility-based models. Note that a previous study reported that open space and recreational land in Hong Kong might increase people’s face-to-face contact rates and thus increase their COVID-19 exposure risk during the pandemic [41]. Therefore, the fear of being exposed to COVID-19 in open space and recreational land may lead to a high level of stress in Hong Kong during the pandemic.

In addition to greenspace, we also examined the associations between PM_2.5_ and noise exposures with depression, anxiety, and stress. We found weak and insignificant associations between PM_2.5_ exposure and depression, anxiety, and stress for the mobility-based models, which only include PM_2.5_ and noise exposures. This result is comparable to previous studies that explore the effects of PM_2.5_ and noise exposures on poor mental health based on the mobility-based approach in the Netherlands: Roberts and Helbich [60] reported that there are no significant linear associations between PM_2.5_ and noise exposures and depression and anxiety. These findings highlight the need for future studies to consider the combined associations between multiple environmental exposures and COVID-19-induced worries about mental health during the pandemic. Further, these results also imply that people may be doubly disadvantaged in poor mental health due to COVID-19-induced worries (e.g., perceived COVID-19 risk, worries about financial hardship, job loss, and family conflicts) and environmental factors (e.g., PM_2.5_) during the pandemic.

Further, we found that the strength and significance of the associations between perceived COVID-19 risk and greenspace exposures with depression, anxiety, and stress differ between residence- and mobility-based exposures. These results are consistent with previous studies, which also reported that results about the health impacts of environmental exposures may be different when using residence- and mobility-based exposures [38,40,44]. Our study further corroborates previous studies by revealing significant differences in the associations between people’s perceived COVID-19 risk and greenspace exposure obtained with a residence-based approach and a mobility-based approach. It underscores the issue of biased environmental exposure assessment when only exposures to the residential environment are taken into account.

### 4.3. Strengths and Limitations

This study is significant because it is one of the first to explore the combined associations between COVID-19-induced worries and multiple environmental exposures with mental health. Further, the use of activity diaries and portable sensors combining real-time noise and air pollutant monitoring allowed for capturing people’s dynamic environmental exposures. In this way, this study aligns with an emerging trend in geography and health research that extends the residence-based approach to the mobility-based approach. Future studies should continue to improve the capability of investigating the combined effects of multiple dynamic social and environmental factors using innovative mobility-based sensors.

The study has a few limitations. First, our survey data were collected from April 2021 to September 2021, which covers the first four waves of the COVID-19 pandemic in Hong Kong. Therefore, the data does not capture the impact of the fifth wave in Hong Kong from December 2021 to April 2022. Although the fifth wave of the COVID-19 outbreak has adversely affected people’s daily lives and health more than before, these differences are unlikely to significantly affect the major conclusions of the study. For instance, our findings have highlighted that the original mental health crisis may spread to different social groups in Hong Kong due to the COVID-19 pandemic, and the fifth wave may accelerate this process.

Second, the activity diary data may be affected by issues of participants’ recall bias. However, we believe that these effects are negligible because we also provided a log sheet to each participant so that they could conveniently and quickly write down the locations and times when they were conducting their daily activities. The information recorded on these log sheets was used to check the activity records in participants’ activity diaries to minimize errors. Future studies would, of course, benefit from using innovative real-time GPS tracking sensors to more accurately capture people’s daily activities and assess their multiple environmental exposures in space and time.

Lastly, our survey only obtained a small sample. Therefore, the generalizability of our results and conclusions might be limited. In addition, our regression results also suggest that the models have relatively low adjusted R^2^ scores (i.e., 0.15 and 0.16 in full models), which indicate that the independent variables (i.e., COVID-19-induced worries and multiple environmental exposures) could explain 15–16% of the variability of the dependent variables (i.e., depression, anxiety, and stress). The possible reasons might include: (1) the small sample size in this study; (2) other covariates (e.g., worries about other issues, comorbid conditions, insomnia, etc.) were not considered. Future studies would benefit from using larger sample sizes with a comprehensive and diverse set of covariates based on similar survey methods to obtain more robust models.

## 5. Conclusions

In conclusion, our study found that people who have a higher perceived COVID-19 risk and are more worried about financial hardship, job loss, and family conflicts have more serious depression, anxiety, and stress symptoms during the pandemic. Meanwhile, people’s exposure to grassland around their daily activity locations is associated with a reduction in depressive symptoms, while exposure to air pollution is associated with worsened depressive symptoms. Residence- and mobility-based perceived COVID-19 risk and greenspace exposure are significantly different, which further translates to differences in the strength and significance of their associations with depression, anxiety, and stress. These associations with depression, anxiety, and stress also differ somewhat depending on people’s demographic attributes. Future research should determine the combined health effects of COVID-19-induced worries and multiple environmental exposures using residence- and mobility-based approaches. These results have important implications for the government in dealing with the public mental health crisis during the COVID-19 pandemic.

## Figures and Tables

**Table 1:** Descriptive statistics of research participants (n = 217).

	Variables	Category	SSP (n = 107)	TSW (n = 110)
			N (%)	N (%)
Socio-demographic status and housing conditions	Sex	Male	44%	46%
		Female	56%	54%
	Age	18–24 years	17%	22%
		25–44 years	47%	47%
		45–65 years	36%	31%
	Education status	With higher education	65%	65%
		without higher education degree	35%	35%
	Monthly household income level (HKD)	Less than 20,000	45%	29%
		20,000–39,999	32%	43%
		40,000 or over	23%	28%
	Employment Status	Housewife	7%	12%
		Employed	82%	74%
		Student	9%	13%
	Marital Status	Married	38%	35%
		Single, widowed, or divorced	62%	65%
	Homeownership	Rented	63%	56%
		Owned	37%	44%
	House type	Social housing	47%	85%
		Private housing	50%	15%
COVID-19 worries	-	Worry about family conflict (Mean (SD))	3.23 (1.30)	2.85 (1.32)
		Worries about financial hardship and job loss (Mean (SD))	7.07 (2.81)	5.55 (2.62)
		Residence-based perceived COVID-19 risk (Mean (SD))	3.37 (0.95)	2.94 (0.77)
		Mobility-based perceived COVID-19 risk (Mean (SD))	2.48 (0.88)	2.50 (0.90)
Outcome	-	Depression and anxiety (Mean (SD))	13.80 (3.82)	13.75 (4.11)
		Stress (Mean (SD))	8.30 (1.67)	8.20 (1.83)

**Table 2:** Residence-based and mobility-based multiple environmental exposures of the sample (n = 217).

Variables	Category	Residence-Based	Mobility-Based	*p*-Value ^a^
Green space	Open Space and Recreational land [Mean (SD)]	0.10 (0.06)	0.12 (0.09)	0.000 ***
	Greenspace [Mean (SD)]	0.09 (0.03)	0.11 (0.07)	0.000 **
PM_2.5_ and noise exposure	PM_2.5_ (ug/m^3^) [Mean (SD)]	-	12.43 (5.57)	-
	Daytime Noise (dBA) [Mean (SD)]	-	63.79 (6.60)	0.000 ***
	Nighttime Noise (dBA) [Mean (SD)]	-	49.15 (6.82)	

Notes: ^a^ Paired sample *t*-test; *** denotes *p* < 0.001; ** denotes *p* < 0.01.

**Table 3:** Associations of COVID-19-induced worries and multiple environmental exposures with people’s depression and anxiety in residence-based and mobility-based linear regression models (n = 217).

Depression and Anxiety						
	Residence-Based			Mobility-Based		
Variables	Model 1	Model 2	Model 3	Model 4	Model 5	Model 6
	Coef. (SE.)	Coef. (SE.)	Coef. (SE.)	Coef. (SE.)	Coef. (SE.)	Coef. (SE.)
PCR ^1^	0.12 * (0.07)	0.13 * (0.06)	0.15 * (0.07)	0.22 *** (0.06)	0.23 *** (0.06)	0.23 ** (0.07)
WFC ^2^	0.11 (0.07)	0.11 (0.08)	0.08 (0.08)	0.10 (0.07)	0.10 (0.08)	0.09 (0.08)
WFHJL ^3^	0.21 ** (0.07)	0.21 ** (0.07)	0.23 ** (0.07)	0.19 ** (0.07)	0.20 ** (0.07)	0.20 ** (0.07)
OSRL ^4^		0.05 (0.06)	0.05 (0.07)		−0.01 (0.06)	0.01 (0.07)
Greenspace		−0.04 (0.07)	−0.05 (0.07)		−0.02 (0.06)	−0.03 (0.07)
PM_2.5_					0.11 * (0.06)	0.09 (0.06)
Daytime Noise					0.01 (0.07)	0.01 (0.07)
Nighttime Noise					0.04 (0.07)	0.04 (0.07)
PCR × Greenspace			0.09 (0.07)			−0.08 (0.06)
WFC × Greenspace			0.02 (0.07)			−0.02 (0.08)
WFHJL × Greenspace			−0.07 (0.07)			−0.03 (0.08)
PCR × OSRL			0.02 (0.08)			0.02 (0.07)
WFC × OSRL			−0.04 (0.08)			−0.08 (0.07)
WFHJL × OSRL			−0.03 (0.08)			−0.05 (0.07)
AIC	608.4	611.6	620.6	599.3	605.6	613.4
Adjusted R^2^	0.13	0.13	0.15	0.13	0.12	0.11

Notes: *** denotes *p* < 0.001; ** denotes *p* < 0.01; * denotes *p* < 0.05. All models control participants’ socio-demographic features, which include age, sex, education, employment, marital status, household income, residence neighborhoods, housing type, homeownership, and working place. ^1^ Perceived COVID-19 risk; ^2^ Worry about family conflict; ^3^ Worry about financial hardship and job loss; ^4^ Open Space and Recreational land.

**Table 4:** Associations of COVID-19-induced worries and multiple environmental exposures with people’s stress in residence-based and mobility-based linear regression models (n = 217).

Stress						
	Residence-Based			Mobility-Based		
Variables	Model 7	Model 8	Model 9	Model 10	Model 11	Model 12
	Coef. (SE.)	Coef. (SE.)	Coef. (SE.)	Coef. (SE.)	Coef. (SE.)	Coef. (SE.)
PCR ^1^	0.08 (0.07)	0.10 (0.06)	0.10 (0.07)	0.18 ** (0.06)	0.17 ** (0.06)	0.17 * (0.07)
WFC ^2^	0.21 ** (0.07)	0.21 ** (0.07)	0.16 * (0.07)	0.20 ** (0.07)	0.20 ** (0.07)	0.21 ** (0.08)
WFHJL ^3^	0.14 * (0.07)	0.15 * (0.07)	0.18 * (0.07)	0.13 * (0.07)	0.14 * (0.07)	0.13 * (0.08)
OSRL ^4^		0.14 * (0.07)	0.14 (0.08)		0.15 * (0.07)	0.15 * (0.07)
Greenspace		0.03 (0.07)	0.01 (0.07)		0.01 (0.06)	0.01 (0.07)
PM_2.5_					0.06 (0.07)	0.05 (0.06)
Daytime Noise					0.02 (0.07)	0.02 (0.07)
Nighttime Noise					−0.01 (0.06)	−0.01 (0.07)
PCR × Greenspace			0.08 (0.07)			−0.03 (0.08)
WFC × Greenspace			0.13 (0.07)			0.10 (0.08)
WFHJL × Greenspace			−0.08 (0.07)			−0.09 (0.06)
PCR × OSRL			0.13 (0.07)			−0.01 (0.08)
WFC × OSRL			0.01 (0.08)			−0.04 (0.07)
WFHJL × OSRL			−0.13 (0.08)			−0.02 (0.07)
AIC	604.7	604.3	606.2	598.3	601.9	611.5
Adjusted R^2^	0.11	0.11	0.13	0.13	0.13	0.12

Notes: ** denotes *p* < 0.01; * denotes *p* < 0.05. All models control participants’ socio-demographic features, which include age, sex, education, employment, marital status, household income, residence neighborhoods, housing type, homeownership, and working place. ^1^ Perceived COVID-19 risk; ^2^ Worry about family conflict; ^3^ Worry about financial hardship and job loss; ^4^ Open Space and Recreational land.

## Data Availability

Data sharing is not applicable to this article due to privacy restrictions.

## Supplementary Materials

The following supporting information can be downloaded at: https://www.mdpi.com/article/10.3390/ijerph20166620/s1, Figure S1. Correlation matrix of (a) residence-based and (b) mobility-based greenspace exposures based on Spearman correlation coefficients. OSRL refers to open space and recreational land; Table S1. Questions and items about people’s depression and anxiety; Table S2. Questions and items about people’s stress; Table S3. Questions and items about people’s COVID-19-related worries; Table S4. Residence-based and mobility-based greenspace exposures (n = 217); Table S5. The performance of the residential-based and mobility-based linear regression models for people’s depression across different groups; Table S6. The performance of the residential-based and mobility-based linear regression models for people’s stress across different groups; Table S7. Results of the linear regression models for participants’ residential neighborhoods. (n = 217); Table S8. Results of the linear regression models for participants’ gender. (n = 217); Table S9. Results of the linear regression models for participants’ marital status. (n = 217); Table S10. Results of the linear regression models for participants’ house type. (n = 217); Table S11. Results of the linear regression models for participants’ household monthly income. (n = 217).

## References

[1] Davies N.G., Kucharski A.J., Eggo R.M., Gimma A., Edmunds W.J., Jombart T., O’Reilly K., Endo A., Hellewell J., Nightingale E.S. (2020). Effects of non-pharmaceutical interventions on COVID-19 cases, deaths, and demand for hospital services in the UK: A modelling study. Lancet Public Health.

[2] Alessandretti L. (2022). What human mobility data tell us about COVID-19 spread. Nat. Rev. Phys.

[3] Bradshaw W.J., Alley E.C., Huggins J.H., Lloyd A.L., Esvelt K.M. (2021). Bidirectional contact tracing could dramatically improve COVID-19 control. Nat. Commun..

[4] Nouvellet P., Bhatia S., Cori A., Ainslie K.E., Baguelin M., Bhatt S., Boonyasiri A., Brazeau N.F., Cattarino L., Cooper L.V. (2021). Reduction in mobility and COVID-19 transmission. Nat. Commun..

[5] Xiong J., Lipsitz O., Nasri F., Lui L.M., Gill H., Phan L., Chen-Li D., Iacobucci M., Ho R., Majeed A. (2020). Impact of COVID-19 pandemic on mental health in the general population: A systematic review. J. Affect. Disord..

[6] Passavanti M., Argentieri A., Barbieri D.M., Lou B., Wijayaratna K., Mirhosseini A.S.F., Wang F., Naseri S., Qamhia I., Tangerås M. (2021). The psychological impact of COVID-19 and restrictive measures in the world. J. Affect. Disord..

[7] Gadermann A.C., Thomson K.C., Richardson C.G., Gagné M., McAuliffe C., Hirani S., Jenkins E. (2021). Examining the impacts of the COVID-19 pandemic on family mental health in Canada: Findings from a national cross-sectional study. BMJ Open.

[8] Posel D., Oyenubi A., Kollamparambil U. (2021). Job loss and mental health during the COVID-19 lockdown: Evidence from South Africa. PLoS ONE.

[9] Pierce M., Hope H., Ford T., Hatch S., Hotopf M., John A., Kontopantelis E., Webb R., Wessely S., McManus S. (2020). Mental health before and during the COVID-19 pandemic: A longitudinal probability sample survey of the UK population. Lancet Psychiatry.

[10] Goularte J.F., Serafim S.D., Colombo R., Hogg B., Caldieraro M.A., Rosa A.R. (2021). COVID-19 and mental health in Brazil: Psychiatric symptoms in the general population. J. Psychiatr. Res..

[11] Généreux M., Schluter P.J., Landaverde E., Hung K.K., Wong C.S., Mok C.P.Y., Blouin-Genest G., O’sullivan T., David M.D., Carignan M.E. (2021). The evolution in anxiety and depression with the progression of the pandemic in adult populations from eight countries and four continents. Int. J. Environ. Res. Public Health.

[12] Jenkins E.K., McAuliffe C., Hirani S., Richardson C., Thomson K.C., McGuinness L., Morris J., Kousoulis A., Gadermann A. (2021). A portrait of the early and differential mental health impacts of the COVID-19 pandemic in Canada: Findings from the first wave of a nationally representative cross-sectional survey. Prev. Med..

[13] Markevych I., Schoierer J., Hartig T., Chudnovsky A., Hystad P., Dzhambov A.M., De Vries S., Triguero-Mas M., Brauer M., Nieuwenhuijsen M.J. (2017). Exploring pathways linking greenspace to health: Theoretical and methodological guidance. Environ. Res..

[14] Kondo M.C., Fluehr J.M., McKeon T., Branas C.C. (2018). Urban green space and its impact on human health. Int. J. Environ. Res. Public Health.

[15] Bratman G.N., Anderson C.B., Berman M.G., Cochran B., De Vries S., Flanders J., Folke C., Frumkin H., Gross J.J., Hartig T. (2019). Nature and mental health: An ecosystem service perspective. Sci. Adv..

[16] Klompmaker J.O., Hoek G., Bloemsma L.D., Wijga A.H., van den Brink C., Brunekreef B., Lebret E., Gehring U., Janssen N.A. (2019). Associations of combined exposures to surrounding green, air pollution and traffic noise on mental health. Environ. Int..

[17] Ribeiro A.I., Triguero-Mas M., Santos C.J., Gómez-Nieto A., Cole H., Anguelovski I., Silva F.M., Baró F. (2021). Exposure to nature and mental health outcomes during COVID-19 lockdown. A comparison between Portugal and Spain. Environ. Int..

[18] Poortinga W., Bird N., Hallingberg B., Phillips R., Williams D. (2021). The role of perceived public and private green space in subjective health and wellbeing during and after the first peak of the COVID-19 outbreak. Landsc. Urban Plan..

[19] Tiako M.J.N., South E., Shannon M.M., McCarthy C., Meisel Z.F., Elovitz M.A., Burris H.H. (2021). Urban residential tree canopy and perceived stress among pregnant women. Environ. Res..

[20] Larson L.R., Mullenbach L.E., Browning M.H., Rigolon A., Thomsen J., Metcalf E.C., Reigner N.P., Sharaievska I., McAnirlin O., D’Antonio A. (2022). Greenspace and park use associated with less emotional distress among college students in the United States during the COVID-19 pandemic. Environ. Res..

[21] da Schio N., Phillips A., Fransen K., Wolff M., Haase D., Ostoić S.K., Živojinović I., Vuletić D., Derks J., Davies C. (2021). The impact of the COVID-19 pandemic on the use of and attitudes towards urban forests and green spaces: Exploring the instigators of change in Belgium. Urban For. Urban Green.

[22] Mouratidis K., Yiannakou A. (2022). COVID-19 and urban planning: Built environment, health, and well-being in Greek cities before and during the pandemic. Cities.

[23] Allard-Poesi F., Matos L.B., Massu J. (2022). Not all types of nature have an equal effect on urban residents’ well-being: A structural equation model approach. Health Place.

[24] Tzivian L., Winkler A., Dlugaj M., Schikowski T., Vossoughi M., Fuks K., Weinmayr G., Hoffmann B. (2015). Effect of long-term outdoor air pollution and noise on cognitive and psychological functions in adults. Int. J. Hyg. Environ. Health.

[25] Dzhambov A.M., Markevych I., Tilov B., Arabadzhiev Z., Stoyanov D., Gatseva P., Dimitrova D.D. (2018). Pathways linking residential noise and air pollution to mental ill-health in young adults. Environ. Res..

[26] van den Bosch M., Meyer-Lindenberg A. (2019). Environmental exposures and depression: Biological mechanisms and epidemiological evidence. Annu. Rev. Public Health.

[27] Recio A., Linares C., Banegas J.R., Díaz J. (2016). Road traffic noise effects on cardiovascular, respiratory, and metabolic health: An integrative model of biological mechanisms. Environ. Res..

[28] Guski R., Schreckenberg D., Schuemer R. (2017). WHO environmental noise guidelines for the European region: A systematic review on environmental noise and annoyance. Int. J. Environ. Res. Public Health.

[29] Shepherd D., Dirks K., Welch D., McBride D., Landon J. (2016). The covariance between air pollution annoyance and noise annoyance, and its relationship with health-related quality of life. Int. J. Environ. Res. Public Health.

[30] Tao Y., Kou L., Chai Y., Kwan M.P. (2021). Associations of co-exposures to air pollution and noise with psychological stress in space and time: A case study in Beijing, China. Environ. Res..

[31] Kwan M.P. (2012). The uncertain geographic context problem. Ann. Assoc. Am. Geogr..

[32] Kwan M.P. (2018). The neighborhood effect averaging problem (NEAP): An elusive confounder of the neighborhood effect. Int. J. Environ. Res. Public Health.

[33] Dewulf B., Neutens T., Lefebvre W., Seynaeve G., Vanpoucke C., Beckx C., Van de Weghe N. (2016). Dynamic assessment of exposure to air pollution using mobile phone data. Int. J. Health Geogr..

[34] James P., Berrigan D., Hart J.E., Hipp J.A., Hoehner C.M., Kerr J., Major J.M., Oka M., Laden F. (2014). Effects of buffer size and shape on associations between the built environment and energy balance. Health Place.

[35] Seidler A., Hegewald J., Seidler A.L., Schubert M., Wagner M., Dröge P., Haufe E., Schmitt J., Swart E., Zeeb H. (2017). Association between aircraft, road and railway traffic noise and depression in a large case-control study based on secondary data. Environ. Res..

[36] Gascon M., Sánchez-Benavides G., Dadvand P., Martínez D., Gramunt N., Gotsens X., Cirach M., Vert C., Molinuevo J.L., Crous-Bou M. (2018). Long-term exposure to residential green and blue spaces and anxiety and depression in adults: A cross-sectional study. Environ. Res..

[37] Klompmaker J.O., Janssen N.A., Bloemsma L.D., Gehring U., Wijga A.H., van den Brink C., Lebret E., Brunekreef B., Hoek G. (2019). Residential surrounding green, air pollution, traffic noise and self-perceived general health. Environ. Res..

[38] Yu H., Russell A., Mulholland J., Huang Z. (2018). Using cell phone location to assess misclassification errors in air pollution exposure estimation. Environ. Pollut..

[39] Helbich M. (2018). Toward dynamic urban environmental exposure assessments in mental health research. Environ. Res..

[40] Huang J., Kwan M.P. (2022). Uncertainties in the assessment of COVID-19 risk: A Study of people’s exposure to high-risk environments using individual-level activity data. Ann. Assoc. Am. Geogr..

[41] Huang J., Kwan M.P. (2023). Associations between COVID-19 risk, multiple environmental exposures, and housing conditions: A study using individual-level GPS-based real-time sensing data. Appl. Geogr..

[42] Yu Z., Liu X. (2023). Spatial variations of the third and fourth COVID-19 waves in Hong Kong: A comparative study using built environment and socio-demographic characteristics. Environ. Plan. B Urban Anal. City Sci..

[43] Kan Z., Kwan M.P., Ng M.K., Tieben H. (2022). The impacts of housing characteristics and built-environment features on mental health. Int. J. Environ. Res. Public Health.

[44] Huang J., Kwan M.P. (2022). Examining the influence of housing conditions and daily greenspace exposure on people’s perceived COVID-19 risk and distress. Int. J. Environ. Res. Public Health.

[45] Huang J., Kwan M.P., Kan Z. (2021). The superspreading places of COVID-19 and the associated built-environment and socio-demographic features: A study using a spatial network framework and individual-level activity data. Health Place.

[46] Kroenke K., Spitzer R.L., Williams J.B., Löwe B. (2009). An ultra-brief screening scale for anxiety and depression: The PHQ–4. Psychosomatics.

[47] Huang J., Kwan M.P., Cai J., Song W., Yu C., Kan Z., Yim S.H.L. (2022). Field evaluation and calibration of low-cost air pollution sensors for environmental exposure research. Sensors.

[48] Choi E.P.H., Hui B.P.H., Wan E.Y.F. (2020). Depression and anxiety in Hong Kong during COVID-19. Int. J. Environ. Res. Public Health.

[49] Robillard R., Saad M., Edwards J., Solomonova E., Pennestri M.H., Daros A., Veissière S.P.L., Quilty L., Dion K., Nixon A. (2020). Social, financial and psychological stress during an emerging pandemic: Observations from a population survey in the acute phase of COVID-19. BMJ Open.

[50] Wilson J.M., Lee J., Fitzgerald H.N., Oosterhoff B., Sevi B., Shook N.J. (2020). Job insecurity and financial concern during the COVID-19 pandemic are associated with worse mental health. Int. J. Occup. Environ. Med..

[51] Zhao S.Z., Wong J.Y.H., Luk T.T., Wai A.K.C., Lam T.H., Wang M.P. (2020). Mental health crisis under COVID-19 pandemic in Hong Kong, China. Int. J. Infect. Dis..

[52] Miconi D., Li Z.Y., Frounfelker R.L., Santavicca T., Cénat J.M., Venkatesh V., Rousseau C. (2021). Ethno-cultural disparities in mental health during the COVID-19 pandemic: A cross-sectional study on the impact of exposure to the virus and COVID-19-related discrimination and stigma on mental health across ethno-cultural groups in Quebec (Canada). BJPsych Open.

[53] Singh R., Subedi M. (2020). COVID-19 and stigma: Social discrimination towards frontline healthcare providers and COVID-19 recovered patients in Nepal. Asian J. Psychiatr..

[54] Fan W., Qian Y., Jin Y. (2021). Stigma, Perceived Discrimination, and Mental Health during China’s COVID-19 Outbreak: A Mixed-Methods Investigation. J. Health Soc. Behav..

[55] Saiz J., Muñoz M., Ausín B., González-Sanguino C., Ángel Castellanos M., Vaquero C., Ugidos C., López-Gómez A. (2021). Effects of COVID-19 lockdown on perceived discrimination and internalized stigma in people with previous mental disorder diagnoses in Spain. Am. J. Orthopsychiatry.

[56] Liu Y., Finch B.K., Brenneke S.G., Thomas K., Le P.D. (2020). Perceived discrimination and mental distress amid the COVID-19 pandemic: Evidence from the understanding America study. Am. J. Prev. Med..

[57] Melton H.C., Belknap J. (2003). He hits, she hits: Assessing gender differences and similarities in officially reported intimate partner violence. Crim. Justice Behav..

[58] Caldwell J.E., Swan S.C., Woodbrown V.D. (2012). Gender differences in intimate partner violence outcomes. Psychol. Violence.

[59] Winstok Z., Straus M.A. (2016). Bridging the two sides of a 30-year controversy over gender differences in perpetration of physical partner violence. J. Fam. Violence.

[60] Roberts H., Helbich M. (2021). Multiple environmental exposures along daily mobility paths and depressive symptoms: A smartphone-based tracking study. Environ. Int..

