# Supplementary material for: How People’s COVID-19 Induced-Worries and Multiple Environmental Exposures Are Associated with Their Depression, Anxiety, and Stress during the Pandemic
Source: Int J Environ Res Public Health. 2023 Aug 21;20(16):6620. doi: 10.3390/ijerph20166620 (PMC10454930; doi:10.3390/ijerph20166620)
Supplement: Supplementary file 1 [file ijerph-20-06620-s001.zip › ijerph-2497121-supplementary.pdf]

Supplementary materials

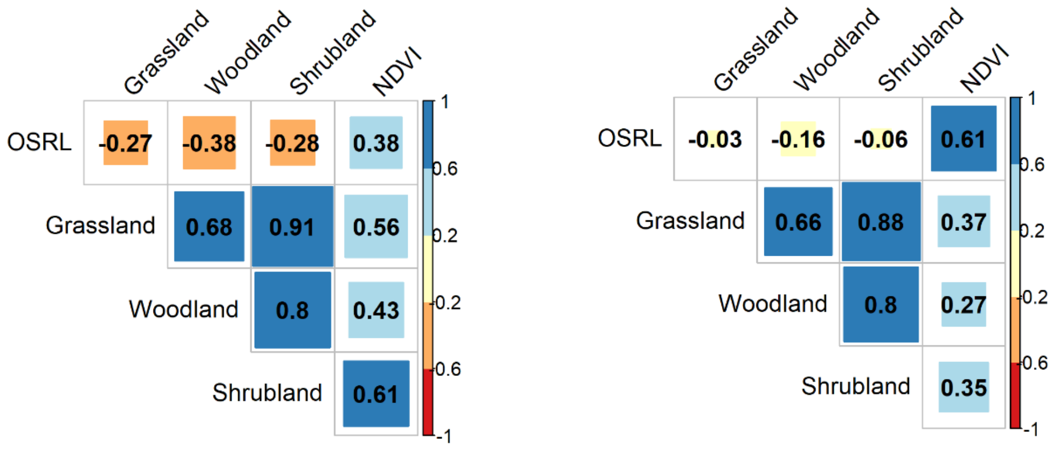

(a) Residence-based (b) Mobility-based

Figure S1. Correlation matrix of (a) residence-based and (b) mobility-based greenspace exposures based on Spearman correlation coefficients. OSRL refers to open space and recreational land.

Table S1. Questions and items about people's depression and anxiety.

Depression and anxiety questions	Items	Scales
Depression and anxiety (PHQ-4): Over the past two weeks, have you been by these problems?	Feeling nervous, anxious, or on edge	1 – 6 ("1" indicates "never", "6" indicates "always)
	Not being able to stop or control worrying	
	Feeling down, depressed, or hopeless	
	Little interest or pleasure in doing things	

Table S2. Questions and items about people's stress.

Stress symptoms questions	Items	Scales	Factor loading [a]
Stress: Over the past year, have you been bothered by these problems?	Distress	1 – 6 ("1" indicates "never", "6" indicates "always")	0.87 *
	Sleep disturbance		0.28
	Fatigue		0.72 *
	Headache		0.47 *

Notes: [a] Exploratory factor analysis; * Factor loadings for each scale item indicate onto which subfactor the scale item loaded.

Table S3. Questions and items about people's COVID-19-related worries.

COVID-19-related worries	Items	Scales
How severe do you think was the transmission of COVID-19 in your residential neighborhood from January 2020?	Residence-based perceived COVID-19 risk	1 – 6 ("1" indicates "very low transmission risk", "6" indicates "the transmission of COVID-19 is severe")
How severe do you think was the transmission of COVID-19 in venues or places you usually visited in one week?	Mobility-based perceived COVID-19 risk	
Over the past year, how has your life been affected by the COVID-19 pandemic?	Worry about job loss	1 – 6 ("1" indicates "never", "6" indicates "always)
	Worry about income reduction	
	Worry about family conflict	

Table S4. Residence-based and mobility-based greenspace exposures (N=217).

**Variables**	Category	Mean	SD	Factor loading [a]
Residence-based greenspace exposures	Grassland	0.03	0.04	0.70 *
	Woodland	0.01	0.01	0.93 *
	Shrubland	0.01	0.02	0.62 *
	NDVI	0.26	0.05	0.60 *
Mobility-based greenspace exposures	Grassland	0.03	0.04	0.68 *
	Woodland	0.01	0.01	0.82 *
	Shrubland	0.01	0.01	0.63 *
	NDVI	0.26	0.05	0.46 *

Notes: [a] Exploratory factor analysis; * Factor loadings for each scale item indicate onto which subfactor the scale item loaded.

Table S5. The performance of the residential-based and mobility-based linear regression models for people's depression across different groups.

Depression				
**Subgroup models**	**Residential-based**		**Mobility-based**	
	AIC	Adjust R^2	AIC	Adjust R^2
SSP	296.81	0.18	294.83	0.19
TSW	325.93	0.13	309.41	0.20
Female	344.91	0.09	343.66	0.20
Male	282.03	0.13	280.04	0.14
Married	222.24	0.21	224.13	0.22
Unmarried	395.51	0.07	394.41	0.12
Social housing	407.50	0.13	404.85	0.12
Private housing	208.39	0.14	213.86	0.10
High income	152.19	0.29	140.82	0.42
Low income	458.74	0.15	441.34	0.13

Table S6. The performance of the residential-based and mobility-based linear regression models for people's stress across different groups.

Stress				
**Subgroup models**	**Residential-based**		**Mobility-based**	
	AIC	Adjust R^2	AIC	Adjust R^2
SSP	292.90	0.21	293.20	0.24
TSW	317.49	0.12	311.11	0.13
Female	348.30	0.04	343.42	0.09
Male	274.94	0.20	267.37	0.24
Married	230.12	0.13	224.87	0.21
Unmarried	384.73	0.13	384.71	0.14
Social housing	399.75	0.18	397.12	0.17
Private housing	218.15	0.13	216.08	0.13
High income	162.68	0.11	160.82	0.12
Low income	446.94	0.14	442.72	0.18

Table S7. Results of the linear regression models for participants' residential neighborhoods. (N = 217).

	**Depression**				**Stress**			
	**Residential-based**		**Mobility-based**		**Residential-based**		**Mobility-based**	
Variables	SSP (N=107)	TSW (N=110)	SSP (N=107)	TSW (N=110)	SSP (N=107)	TSW (N=110)	SSP (N=107)	TSW (N=110)
	Coef. (SE)	Coef. (SE)	Coef. (SE)	Coef. (SE)	Coef. (SE)	Coef. (SE)	Coef. (SE)	Coef. (SE)
Greenspace								
Open Space and Recreational land	0.06 (0.10)	0.01 (0.11)	0.07 (0.09)	-0.04 (0.11)	0.11 (0.09)	-0.03 (0.21)	0.12 (0.10)	0.21* (0.10)
Greenspace	-0.05 (0.09)	0.01 (0.12)	-0.10 (0.15)	-0.05 (0.106)	-0.13 (0.10)	-0.27 (0.15)	-0.08 (0.11)	-0.08 (0.09)
COVID-19 risk and worries								
Perceived COVID-19 risk	0.21* (0.10)	0.14 * (0.09)	0.19 * (0.11)	0.21* (0.09)	0.07 (0.09)	0.13 (0.10)	0.05 (0.10)	0.19* (0.09)
Worry about family conflict	0.24 * (0.11)	0.06 (0.11)	0.25 * (0.10)	0.02 (0.10)	0.21* (0.11)	0.20 * (0.11)	0.21* (0.11)	0.20* (0.11)
Worries about financial hardship and job loss	0.27 * (0.11)	0.16* (0.11)	0.23* (0.11)	0.26* (0.11)	0.29** (0.11)	0.10 (0.11)	0.30* (0.12)	0.09 (0.10)
$PM_{2.5}$ and noise exposures								
$PM_{2.5}$	-	-	0.07 (0.10)	0.15 (0.10)	-	-	0.02 (0.10)	0.11 (0.10)
Daytime noise	-	-	0.03 (0.10)	-0.02 (0.10)	-	-	0.05 (0.10)	0.03 (0.09)
Nighttime noise	-	-	0.06 (0.11)	0.07 (0.10)	-	-	-0.11 (0.10)	0.07 (0.10)

Notes: ** denotes $p < 0.01$; * denotes $p < 0.05$. All models control participants' socio-demographic features, which include age, gender, education, employment, marital status, household income, residence neighborhoods, housing type, homeownership, and working place.

Table S8. Results of the linear regression models for participants' gender. (N = 217).

	**Depression**				**Stress**			
	**Residential-based**		**Mobility-based**		**Residential-based**		**Mobility-based**	
Variables	Female (N=119)	Male (N=98)	Female (N=119)	Male (N=98)	Female (N=119)	Male (N=98)	Female (N=119)	Male (N=98)
	Coef. (SE)	Coef. (SE)	Coef. (SE)	Coef. (SE)	Coef. (SE)	Coef. (SE)	Coef. (SE)	Coef. (SE)
Greenspace								
Open Space and Recreational land	0.01 (0.10)	0.07 (0.11)	0.02 (0.14)	-0.06 (0.11)	0.17 (0.10)	0.11 (0.10)	0.24 * (0.09)	0.01 (0.10)
Greenspace	-0.11 (0.09)	-0.12 (0.15)	-0.11 (0.09)	-0.09 (0.10)	-0.02 (0.10)	-0.17 (0.14)	-0.14 (0.09)	-0.20* (0.10)
COVID-19 risk and worries								
Perceived COVID-19 risk	0.08 (0.09)	0.31** (0.11)	0.24* (0.09)	0.31** (0.10)	0.14 (0.10)	0.15 (0.10)	0.13 (0.10)	0.25* (0.10)
Worry about family conflict	0.11 (0.11)	0.05 (0.12)	0.10 (0.10)	0.07 (0.12)	0.19* (0.11)	0.22* (0.11)	0.19 (0.11)	0.23* (0.11)
Worries about financial hardship and job loss	0.26** (0.10)	0.22* (0.11)	0.27** (0.11)	0.22* (0.12)	0.08 (0.11)	0.27** (0.11)	0.07 (0.10)	0.26* (0.11)
$PM_{2.5}$ and noise exposures								
$PM_{2.5}$	-		0.01 (0.09)	0.22* (0.11)	-	-	-0.04 (0.09)	0.16* (0.10)
Daytime noise	-		-0.02 (0.09)	0.01 (0.11)	-	-	-0.11 (0.10)	-0.06 (0.10)
Nighttime noise	-		-0.01 (0.10)	0.11 (0.11)	-	-	0.14 (0.09)	0.19* (0.10)

Notes: ** denotes $p < 0.01$; * denotes $p < 0.05$. All models control participants' socio-demographic features, which include age, gender, education, employment, marital status, household income, residence neighborhoods, housing type, homeownership, and working place.

Table S9. Results of the linear regression models for participants' marital status. (N = 217).

	Depression				Stress			
	**Residential-based**		**Mobility-based**		**Residential-based**		**Mobility-based**	
Variables	Married (N=80)	Unmarried (N=137)	Married (N=80)	Unmarried (N=137)	Married (N=80)	Unmarried (N=137)	Married (N=80)	Unmarried (N=137)
	Coef. (SE)	Coef. (SE)	Coef. (SE)	Coef. (SE)	Coef. (SE)	Coef. (SE)	Coef. (SE)	Coef. (SE)
Greenspace								
Open Space and Recreational land	0.04 (0.09)	0.03 (0.09)	0.06 (0.12)	-0.04 (0.09)	0.18 (0.08)	0.18 (0.08)	0.25* (0.12)	0.12 (0.08)
Greenspace	-0.13 (0.09)	-0.14 (0.09)	-0.01 (0.10)	-0.08 (0.09)	-0.10 (0.08)	-0.11 (0.09)	-0.05 (0.11)	-0.04 (0.09)
COVID-19 risk and worries								
Perceived COVID-19 risk	0.06 (0.11)	0.17* (0.09)	0.19* (0.11)	0.19* (0.08)	0.07 (0.12)	0.14 (0.08)	0.05 (0.11)	0.16* (0.08)
Worry about family conflict	0.13 (0.15)	0.04 (0.09)	0.06 (0.15)	0.04 (0.09)	0.27* (0.15)	0.17* (0.09)	0.25* (0.15)	0.18* (0.09)
Worries about financial hardship and job loss	0.43** (0.14)	0.19* (0.09)	0.46** (0.14)	0.17* (0.09)	0.26* (0.15)	0.21** (0.08)	0.32* (0.16)	0.21** (0.09)
$PM_{2.5}$ and noise exposures								
$PM_{2.5}$	-	-	0.05 (0.12)	0.19* (0.08)	-	-	0.09 (0.12)	0.15* (0.08)
Daytime noise	-	-	-0.01 (0.11)	0.06 (0.09)	-	-	-0.25* (0.11)	0.12 (0.09)
Nighttime noise	-	-	-0.06 (0.12)	0.05 (0.09)	-	-	0.02 (0.11)	0.03 (0.08)

Notes: ** denotes $p < 0.01$; * denotes $p < 0.05$. All models control participants' socio-demographic features, which include age, gender, education, employment, marital status, household income, residence neighborhoods, housing type, homeownership, and working place.

Table S10. Results of the linear regression models for participants' house type. (N = 217).

	**Depression**				**Stress**			
	**Residential-based**		**Mobility-based**		**Residential-based**		**Mobility-based**	
Variables	Social house (N=144)	Non-social house (N=73)	Social house (N=144)	Non-social house (N=73)	Social house (N=144)	Non-social house (N=73)	Social house (N=144)	Non-social house (N=73)
	Coef. (SE)	Coef. (SE)	Coef. (SE)	Coef. (SE)	Coef. (SE)	Coef. (SE)	Coef. (SE)	Coef. (SE)
Greenspace								
Open Space and Recreational land	0.02 (0.08)	0.17 (0.13)	-0.01 (0.09)	-0.03 (0.13)	0.20* (0.08)	0.06 (0.12)	0.18* (0.08)	0.02 (0.13)
Greenspace	-0.02 (0.09)	-0.13 (0.11)	-0.05 (0.08)	-0.12 (0.12)	-0.06 (0.08)	-0.01 (0.12)	-0.07 (0.08)	-0.18 (0.12)
COVID-19 risk and worries								
Perceived COVID-19 risk	0.10 (0.08)	0.27* (0.12)	0.22** (0.08)	0.27* (0.12)	0.08 (0.08)	0.10 (0.13)	0.15* (0.08)	0.24* (0.13)
Worry about family conflict	0.09 (0.09)	0.19 (0.12)	0.07 (0.09)	0.21 (0.13)	0.26** (0.08)	0.18 (0.15)	0.24** (0.09)	0.12 (0.15)
Worries about financial hardship and job loss	0.20* (0.09)	0.21 (0.14)	0.24** (0.09)	0.20 (0.15)	0.21* (0.09)	0.13 (0.15)	0.12 (0.08)	0.11 (0.16)
$PM_{2.5}$ and noise exposures								
$PM_{2.5}$	-	-	0.13* (0.07)	0.12 (0.12)	-	-	0.09 (0.07)	0.03 (0.13)
Daytime noise	-	-	-0.01 (0.08)	-0.04 (0.13)	-	-	0.09 (0.08)	-0.11 (0.12)
Nighttime noise	-	-	0.06 (0.08)	0.06 (0.12)	-	-	0.02 (0.08)	0.01 (0.13)

Notes: ** denotes $p < 0.01$; * denotes $p < 0.05$. All models control participants' socio-demographic features, which include age, gender, education, employment, marital status, household income, residence neighborhoods, housing type, homeownership, and working place.

Table S11. Results of the linear regression models for participants' household monthly income. (N = 217).

	**Depression**				**Stress**			
	**Residential-based**		**Mobility-based**		**Residential-based**		**Mobility-based**	
Variables	High income (N=56)	Low income (N=161)	High income (N=56)	Low income (N=161)	High income (N=56)	Low income (N=161)	High income (N=56)	Low income (N=161)
	Coef. (SE)	Coef. (SE)	Coef. (SE)	Coef. (SE)	Coef. (SE)	Coef. (SE)	Coef. (SE)	Coef. (SE)
Greenspace								
Open Space and Recreational land	0.18 (0.15)	0.02 (0.08)	0.30 (0.16)	-0.02 (0.09)	0.23* (0.15)	0.12 (0.08)	0.35* (0.18)	0.15* (0.08)
Greenspace	-0.04 (0.20)	0.01 (0.08)	-0.30* (0.15)	-0.07 (0.08)	-0.07 (0.18)	0.03 (0.09)	-0.36* (0.17)	-0.08 (0.08)
COVID-19 risk and worries								
Perceived COVID-19 risk	0.11 (0.13)	0.17* (0.08)	0.01 (0.12)	0.30*** (0.08)	0.02 (0.16)	0.16* (0.07)	0.12 (0.15)	0.21** (0.08)
Worry about family conflict	0.07 (0.13)	0.15* (0.09)	0.03 (0.13)	0.14 (0.09)	0.23* (0.16)	0.25** (0.08)	0.03 (0.15)	0.27** (0.09)
Worries about financial hardship and job loss	0.51** (0.13)	0.15* (0.10)	0.50** (0.12)	0.16* (0.09)	0.22* (0.17)	0.14 (0.09)	0.51** (0.18)	0.12 (0.09)
$PM_{2.5}$ and noise exposures								
$PM_{2.5}$	-	-	0.31* (0.14)	0.04 (0.07)	-	-	0.11 (0.15)	0.11 (0.07)
Daytime noise	-	-	-0.07 (0.12)	0.03 (0.08)	-	-	-0.01 (0.14)	0.05 (0.08)
Nighttime noise	-	-	0.09 (0.13)	0.01 (0.08)	-	-	0.15 (0.15)	-0.05 (0.08)

Notes: *** denotes $p < 0.001$; ** denotes $p < 0.01$; * denotes $p < 0.05$. All models control participants' socio-demographic features, which include age, gender, education, employment, marital status, household income, residence neighborhoods, housing type, homeownership, and working place.
